# Bridging the Science Practices Gap: Analyzing Laboratory Materials for Their Opportunities for Engagement in Science Practices

**DOI:** 10.1021/acs.jchemed.4c00744

**Published:** 2025-02-11

**Authors:** Andrea L. Van Wyk, Ardith Bhinu, Kimberley A. Frederick, Marya Lieberman, Renée S. Cole

**Affiliations:** † Department of Chemistry, University of Iowa, Iowa City, Iowa 52242, United States; ‡ Department of Chemistry, Skidmore College, Saratoga Springs, New York 12866, United States; § Department of Chemistry and Biochemistry, University of Notre Dame, Notre Dame, Indiana 46556, United States

**Keywords:** Second-Year Undergraduate, Laboratory Instruction, Inquiry-Based/Discovery Learning, Professional Development, Chemistry Education Research

## Abstract

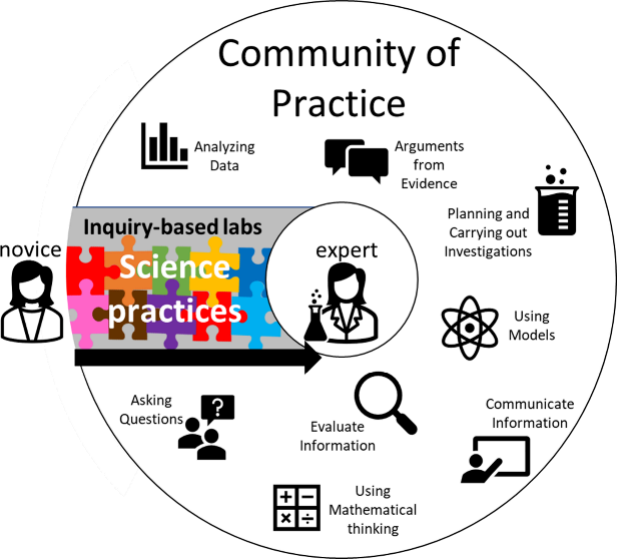

Laboratory courses provide a unique environment for students to engage in the practices of scientists and develop the skills necessary for a career in science. Research has indicated that inquiry-based laboratory activities, which allow students to make key decisions throughout the laboratory experiment, provide opportunities to develop these necessary skills and practices. This study focuses on characterizing laboratory experiments used in analytical chemistry courses for their level of inquiry and opportunities for engagement in scientific practices. Data for this analysis was collected from participants of the MICRO project, a project designed to assist instructors in adopting inquiry-based laboratory experiments. Laboratory experiments used by instructors prior to their involvement in the project and then during the semester of implementation of the inquiry-based laboratory experiments were analyzed for their level of inquiry and opportunities for engagement in scientific practices. We found that, despite an increase in the level of inquiry of laboratory materials, there was not an increase in prompted opportunities to engage students in all of the science practices. We also found that certain science practices were more likely to be prompted in certain sections of the laboratory materials, such as through the pre-laboratory and post-laboratory questions.

## Introduction

What skills or attributes are characteristic of a successful scientist? When employers and graduate/professional programs are asked this question, the answer lies not in a domain specific knowledge base but rather in the skills with which they are proficient. Such skills include problem solving, thinking critically, evaluating data, forming arguments, and communicating data.^1−5^ These skills are valuable for all individuals and beneficial to strengthening the workplace and tackling the worldwide challenges we are facing. There have been several reports that highlighted a lack of preparation and an apparent skills gap of STEM graduates entering the workforce,^6−8^ including several specifically focused on chemistry.^1−4^ In 2012 the National Research Council released *A Framework for K-12 Science Education*, which outlined three dimensions of science learning: disciplinary core ideas, crosscutting concepts, and science and engineering practices.^9^ This shifted science curricula from solely focusing on scientific concepts to the integration of scientific concepts and practices through the Next Generation Science Standards (NGSS) for K-12 education.^10^ These practices are listed in Table 1.

**Table 1:** List of Science Practices defined by *The Framework*(9)

Science Practices
Asking questions
Developing and using models
Planning and carrying out investigations
Analyzing and interpreting data
Using mathematics and computational thinking
Constructing explanations
Engaging in argument from evidence
Obtaining, evaluating, and communicating information

Within chemistry higher education, accrediting bodies such as the American Chemical Society Committee on Professional Training (ACS-CPT)^11,12^ and Royal Society of Chemistry (RSC)^13^ have emphasized the need to develop science practices, specifically through time spent in the laboratory, mandating at least 350 h (CPT) or 300 h (RSC) of laboratory work integrated into the curriculum to obtain a Bachelor’s degree in chemistry. The 2023 Revised ACS Guidelines places greater emphasis on scientific practices, particularly in the context of skills learned in laboratory courses.^12^

### Laboratory Initiatives Designed to Engage Students in Science Practices

Laboratory learning has historically relied on the use of “cookbook” style laboratory experiments.^14,15^ These types of laboratory approaches are not representative of the work of scientists, as they often do not give students opportunities to engage in the types of skills and practices of a working scientist. In contrast, inquiry-based laboratory experiments and research experiences allow students to get a more accurate understanding of what scientists do and engage in skills and science practices in order to develop proficiency with them prior to entering the workforce.^14,15^ While introductory chemistry courses may use more prescriptive “cookbook” style laboratory experiments to develop proficiency with techniques or laboratory skills, scaffolding laboratory experiences across chemistry curriculum that engage students in higher levels of inquiry laboratory experiments and more authentic research are instrumental in closing the skills gap.^16−18^

Throughout the years, there have been several laboratory initiatives to introduce inquiry and research experiences into the laboratory curriculum. Science Writing Heuristic, Argument Driven Inquiry, and Problem-Based Learning are three such inquiry-based laboratory models where students are engaged in discovery.^19−21^ These types of approaches have been shown to engage students in several science practices.^22−25^ Most chemistry programs also seek to provide opportunities for students to engage in authentic research experiences. There exist many different avenues for undergraduate research experiences (UREs), but as of late, a number of course-based undergraduate research experiences (CUREs) have emerged, embedding these same types of research experiences in the laboratory course setting. Research has shown a number of benefits to students who engage in UREs and CUREs, including learning to think like a scientist, increased scientific skills, increased self-efficacy, and improved retention in STEM degrees.^26−32^ While there are some trade-offs in time and content coverage, these types of inquiry-based laboratory experiments and authentic research experiences are instrumental in allowing students to develop proficiency with the skills and science practices they will need.

### Previous Work to Characterize Laboratory Experiments for Opportunities to Engage in Science Practices

Previous work has developed an instrument, the Three-Dimensional Learning Assessment Protocol (3D-LAP), which evaluates assessments for their opportunities to engaged students across all three dimensions of science learning, laid out by NGSS.^10,33^ While this instrument was designed to examine assessments for their opportunities to engage students in science practices, Carmel and colleagues adapted the science practices dimension of the 3D-LAP to analyze laboratory experiments.^25^ When they applied the modified 3D-LAP to two different General Chemistry laboratory curricula, one focused on traditional, cookbook style laboratory experiments and the other on a project-based laboratory curriculum, they found that the project-based laboratory curriculum had more opportunities to engage in science practices than the traditional laboratory curriculum.^25^ Others have found this trend to hold true with other inquiry-based styles of learning in the laboratory including Science Writing Heuristic^24^ and Argument Driven Inquiry.^34^

### Objectives of This Research Study

To better understand the opportunities students have to engage with science practices in laboratory courses beyond general chemistry, we sought to examine analytical chemistry course materials for their opportunities to engage students with science practices. The analytical chemistry curriculum was of interest because it has a long history of relying on cookbook style laboratory experiments due to a heavy reliance on accuracy and precision. Laboratory materials were collected as part of a professional development project designed to encourage analytical chemistry faculty at various institutions to adopt an inquiry-based laboratory approach. The research questions guiding this work are

1. What opportunities are provided to students to engage in science practices in analytical chemistry laboratory experiments?
2. Are there aspects of laboratory materials that provide opportunities to engage in certain science practices more than others?

## THEORETICAL FRAMEWORKS

The theoretical frameworks guiding this research study are situated learning and constructive alignment. Situated learning is a learning theory originating from the works of Brown et al.^35^ and Lave and Wenger^36−38^ that describes learning as situated in a specific context and social environment. Previous learning theories were purely cognitive, where knowledge is an entity that can be gained or transferred between decontextualized individuals.^39^ Situated learning, however, considers learning to be a generative process where knowledge is constructed while the individual interacts with the context and other individuals in the environment.^39^ Lave and Wenger created the term “legitimate peripheral participation” to describe how learning occurs through this lens. They define learning as the process of increased participation within a community of practice (CoP).^37^ A CoP is a group of individuals who share common goals, beliefs, and norms of practices.^37^ When a newcomer joins a CoP they are on the periphery of the CoP. As they start to participate in the community and interact with other members of the community, they learn about goals, techniques, and language of the community, becoming more proficient and invested within it.

For our study, students taking chemistry courses, particularly those working on a degree in chemistry, are integrating into a CoP that is a part of the discipline of chemistry itself. Within the discipline of chemistry, there are established resources such as the periodic table, common practices such as conducting experiments and constructing arguments, and language such as nomenclature and vocabulary that students are becoming familiar with. As a student moves through a chemistry major’s program of study, they should become integrated into the community and develop proficiency with the knowledge and skills of the discipline. In our study, we are specifically investigating the opportunities that students have to engage in science practices through laboratory activities. For the purposes of this study, we define engagement as behavioral or cognitive depending on the nature of the specific science practice.

The other theoretical framework guiding our work is constructive alignment. Constructive alignment was introduced as a model for creating or modifying curriculum to align with the intended learning outcomes to ensure that students are getting opportunities to practice and be assessed on those learning outcomes.^40,41^ Within constructive alignment there are three elements: intended learning objectives, tasks, and assessment.^40,41^ For our work, the intended learning objectives are the science and engineering practices created by NGSS.^9,10^ Developing proficiency with these practices is emphasized as a goal of chemistry education by a number of accrediting bodies.^12,13^ The tasks in our study are the laboratory activities that students complete and opportunities for students to engage in science practices prompted by those materials. Lastly, assessment in this study is the deliverable for the laboratory activity, usually a laboratory report or presentation. When these three elements of constructive alignment are misaligned, students will be achieving a set of learning objectives that are different than the set intended and not achieving the intended set of learning objectives. If our goal is for students to be proficient with science practices when they leave a chemistry degree program but they are not given opportunities along the way to engage in science practices, it is unlikely that they will leave the program proficient with these practices vital to being an acting scientist.

## Methods

### Participants and Settings

The laboratory materials analyzed for this study were collected as part of the MICRO (Making Introductory Chemistry Real while Online) project.^42,43^ The project aimed to provide inquiry-based laboratory experiences that used paper-based microfluidic technology to conduct traditional analytical chemistry experiments safely at home or in a laboratory setting. There were two cohorts of faculty who participated in the MICRO project. Cohort 1 consisted of 20 faculty from 19 institutions during the 2020–2021 academic year. Two faculty members from one institution team-taught an analytical course and implemented the MICRO laboratory experiments together. Of those 19 participating institutions, 16 (84.2%) consented to having their data used for this study and 13 submitted a complete data set of laboratory experiments for analysis. Cohort 2 consisted of 19 faculty from 18 institutions during the 2021–2022 academic year, with two faculty team-teaching a course. Of those 18 participating institutions, 9 (50%) institutions consented to having their data used for this study and 4 submitted a complete data set of laboratory experiments for analysis. Faculty in both cohorts taught at a variety of institutions, ranging from baccalaureate colleges to doctoral universities.

### Data Collection

After faculty applied and were invited to participate in the MICRO project, they were asked to submit laboratory materials that they used in their analytical chemistry course the last time they taught it. This set of laboratory experiments served as the pre-implementation data set. During the summer faculty engaged with the MICRO community through a 3-day workshop, primarily focusing on inquiry-based laboratory learning and details of the MICRO laboratory experiments. At the workshop, science practices were discussed, but no formal training was presented to participants. After the workshop, faculty implemented 2–4 of the MICRO laboratory experiments in their analytical chemistry course. At the conclusion of the semester of implementation, faculty uploaded all of their laboratory materials used that semester, including the MICRO laboratory materials since instructors were encouraged to modify them for their unique learning environments. This served as the post-implementation data set.

### Data Analysis: Determining the Opportunities to Engage in Science Practices

The opportunities for engagement in science practices for each of the laboratory experiments used by participants of the MICRO project were analyzed using the version of the 3D-LAP modified for laboratory materials.^25,33^ Using the criteria laid out for each science practice in the modified 3D-LAP, we developed a complete codebook.^25^ The modified 3D-LAP instrument defined each science practice by two to four criteria. For all but one practice (the engineering practice), the first criterion is the same; it establishes the “event, observation, phenomenon, data, scenario, or model” that the laboratory focuses on. The criteria tend to build on themselves with the last criterion often being a prompt for students to provide an interpretation, justification, or reasoning. When looking at the criteria provided for each science practice in the 3D-LAP, there were often two ways a criterion could be met. For instance, for *developing and using models* (SP 2), criterion 2 states “Question gives a representation or asks students to construct a representation.” In thinking about the level of independence and competence a student has with a science practice, we decided that it was important to distinguish between the instances where students were given a representation and the instances where they have to construct a representation. To accomplish this, we separated the criterion into two separate criteria labeled *a* and *b* as seen in the example in Table 2 and in the codebook included in the Supporting Information. To determine whether a laboratory experiment had all the criteria needed to provide a complete prompt to engage in a science practice, the presence of either *a* or *b* was sufficient.

**Table 2:** Example of Original Criteria from the 3D-LAP^33^ and Separated Criteria in Our Modified Codebook

3D-LAP Original Criteria	Our Separated Criteria
*Developing and using models* (Criterion 2): Question gives a representation or asks student to construct a representation	*Developing and using models* (Criterion 2a): Laboratory experiment gives a representation/model.
	*Developing and using models* (Criterion 2b): Laboratory experiment asks student to construct a representation.

Additionally, we clarified some language and definitions as needed to have consistency and specificity when coding laboratory materials. For the practice *developing and using models* (SP 2), we clarified the definitions of representation and model using the framework laid out by Zwickl et al.^44^ They established that external representations are a key component of a model and can be computational, mathematical, verbal, diagrammatic, or graphical. The other science practice that was clarified was that of *communicating information*.^25^ The second criterion for that practice was “Activity asks students to communicate their findings to an audience beyond the instructor in a way that could be understood by the general public.” We considered the different forms of communication a chemist may engage in and decided that it would be important to consider not only the general science communication originally defined by Carmel et al. but also the skill of technical writing to other experts in chemistry.^25^ To address this, we broke this criterion into two different criteria, as seen in Table 3. The presence of either *a* or *b* was sufficient for satisfying criterion 2.

**Table 3:** Original Criterion 2 from Carmel et al.’s Modified 3D-LAP^25^ and Our Modified Criteria for *Communicate Information*

3D-LAP Original Criteria	Our Separated Criteria
*Communicate information* (Criterion 2): Laboratory experiment asks students to communicate their findings to an audience beyond the instructor in a way that could be understood by the public.	*Communicate information* (Criterion 2a): Laboratory experiment asks students to communicate their findings to another scientist.
	*Communicate information* (Criterion 2b): Laboratory experiment asks students to communicate their findings to a general public audience.

To analyze the materials, we recorded which criteria for each of the nine science practices were present in the laboratory activity and assigned a category from Table 4 for that science practice.

**Table 4:** Categories for the Extent to Which the 3D-LAP Criteria for Each Science Practice Were Prompted within a Laboratory Activity

Category	Definition for Practices with 4 Criteria (SP 2, 4, 6, and 8)	Definition for Practices with 3 Criteria (SP 3, 5, and 7)	Definition for Practices with 2 Criteria (SP 1 and 9)
Complete prompting	All criteria for the prompting of a science practice are met	All criteria for the prompting of a science practice are met	All criteria for the prompting of a science practice are met
Task only	All but the last criterion, the prompting for a justification, are met	All but the last criterion, the prompting for a justification, are met	N/A
Partial task only	2 of 4 criteria are met, only part of the task	N/A	N/A
Base criteria	Only the first criterion for the prompting of a science practice is met	Only the first criterion for the prompting of a science practice is met	Only the first criterion for the prompting of a science practice is met
Absent	All criteria are absent	All criteria are absent	All criteria are absent

For example, if the first two criteria for *analyzing and interpreting data* (SP 4) were present in a laboratory experiment, the laboratory experiment would be classified as “partial task only” since this science practice is defined by 4 criteria and only the base criteria and one criterion (out of two) for the task was prompted in the materials. However, for a science practice such as *planning and carrying out investigations* (SP 3), if the first two criteria were present, since it is only defined by 3 criteria, this laboratory experiment would be considered “task only” as it contained the prompts for the task but not a prompt for providing a justification (last criterion). Using this scheme allowed us to categorize laboratory materials that may not have a complete opportunity to engage students in a given science practice but include some aspects, such as “task only” or “partial task only”, in which case modifications can be suggested to faculty to improve the activities. It is important to note, however, that the presence of just the base criteria for a science practice was not enough evidence to indicate any partial prompted opportunity of engagement in a science practice due to its similarity across all of the science practices.

### Data Analysis: Determining Levels of Inquiry

To determine if there were any trends in laboratory materials regarding the opportunities that exist for students to engage in science practices and the laboratory experiment’s level of inquiry, each laboratory experiment was coded for its level of inquiry. This work was reported earlier in Van Wyk et al.^45^ A modified version of a levels of inquiry rubric designed by Bruck et al.^14,45^ was applied to each laboratory experiment to categorize it on a scale of 0 (confirmation laboratory) to 3 (authentic inquiry laboratory), as seen in Table 5. This rubric differs from that of Bruck et al. primarily through the use of half levels and partially provided and half provided laboratory components. A “half provided” laboratory component is when a significant (typically about 50%) portion of the laboratory component is left up to the student. The “partially provided” component is when students must make decisions but are still provided with 50% or more of that component.

**Table 5:** Levels of Inquiry Rubric Applied to Each Laboratory Experiment Modified from Bruck et al.^14,45^

Lab Component	Level 0: Confirmation	Level 0.5: Structured Inquiry	Level 1: Guided Inquiry	Level 1 Alternative	Level 1.5	Level 2: Open Inquiry	Level 2 Alternative	Level 2.5	Level 3: Authentic Inquiry
Problem/Question	Provided	Provided	Provided	Provided	Provided	Provided	Provided	Provided	Not provided
Theory/Background	Provided	Provided	Provided	Provided	Provided	Provided	Partially provided	Not provided	Not provided
Procedure/Design	Provided	Provided	Provided	Partially provided	Half provided	Not provided	Partially provided	Not provided	Not provided
Results analysis	Provided	Provided	Not provided	Partially provided	Not provided	Not provided	Not provided	Not provided	Not provided
Results communication	Provided/Partially provided	Provided/Partially provided	Partially provided/Not provided	Partially provided/Not provided	Partially provided/Not provided	Partially provided/Not provided	Partially provided/Not provided	Partially provided/Not provided	Partially provided/Not provided
Conclusions	Provided	Not provided	Not provided	Not provided	Not provided	Not provided	Not provided	Not provided	Not provided

### Coding and IRR Process

Initially, laboratory experiments representing different levels of inquiry were analyzed with the modified 3D-LAP coding scheme to determine if the coding scheme was appropriate for all types of laboratory experiments, and with the assumption that certain science practices were more likely to occur in higher inquiry laboratory experiments, this would ensure practice coding with as many science practices as possible. Negotiated agreement between two coders (A.L.V.W. and A.B.) was used in this stage to discuss differences in coding, and the codebook was further clarified based on conversations. Once coding was consistent and no further changes to the codebook were needed, all 244 laboratory experiments were divided among the two coders (A.L.V.W. and A.B.) and coded. The complete codebook is included in the Supporting Information. Interrater reliability measures were calculated using Gwet’s AC1, an adapted version of Kohen’s kappa, for 20% of the laboratory experiments coded by both coders (*n* = 49) throughout the coding process.^46^ Interrater reliability was established from the 20% of laboratory experiments with a κ = 0.78 indicating upper moderate reliability.^47^

## Results and Discussion

In a previous study investigating the level of inquiry of these same laboratory materials it was found that there was a shift to higher percentages of inquiry-based laboratory materials when comparing faculty’s initial laboratory materials with their laboratory materials used during the semester of implementation, even when excluding the MICRO laboratory experiments.^45^ This indicated participants’ adoption of inquiry-based instruction and provided evidence that they revised some laboratory materials to be higher in inquiry.^45^ One of the selling points of higher inquiry laboratory experiments is that they are said to be more authentic to the work of scientists and get students involved in the practices of scientist. One may assume that moving toward higher levels of inquiry laboratory materials would thus provide more opportunities to engage in science practices. This relationship has been demonstrated by Carmel et al., who showed that a higher-inquiry, project-based general chemistry curriculum provided more opportunities to engage students in science practices than a traditional “cookbook” style general chemistry laboratory curriculum.^25^

Given the increased adoption of inquiry-based laboratory experiments and prior work indicating a possible connection between higher inquiry laboratory materials providing more opportunities to engage in science practices, we expected to see similar increases in the prompted opportunities for students to engage in science practices when comparing materials from pre-implementation and the semester of engagement. The detailed results of this analysis are listed in the Supporting Information. Since the MICRO laboratory experiments emphasized opportunities to engage in science practices (Figure S2), the incorporation of 2–4 MICRO laboratory experiments did result in more prompted opportunities to engage in science practices (Figure S3). However, when the MICRO experiments were excluded from the post-implementation laboratory materials to determine changes in prompted opportunities due to faculty modifying their materials, we saw relatively no differences (Figure S4).

This raised the question of why intentional changes to increase the level of inquiry of a laboratory experiment did not result in increases in prompted opportunities to engage in science practices as previous work had indicated. We sought to further investigate this assumed connection between inquiry and science practices by comparing the levels of inquiry of the laboratory materials (reported in Van Wyk et al.^45^) to the prompted opportunities to engage students in science practices.

### Levels of Inquiry vs Opportunities to Engage in Science Practices

To investigate possible trends with the levels of inquiry of a laboratory experiment and prompted opportunities for students to engage in science practices, we sorted the laboratory experiments by their level of inquiry. We wanted to compare all the distinct laboratory materials, so we included the 8 MICRO laboratory experiments, pre-implementation laboratory materials, and non-MICRO post-implementation laboratory materials so there would not be duplicates. The analysis of laboratory materials for the relationship between the level of inquiry and opportunities to engage students in science practices revealed different patterns for different science practices.

**Figure 1:**
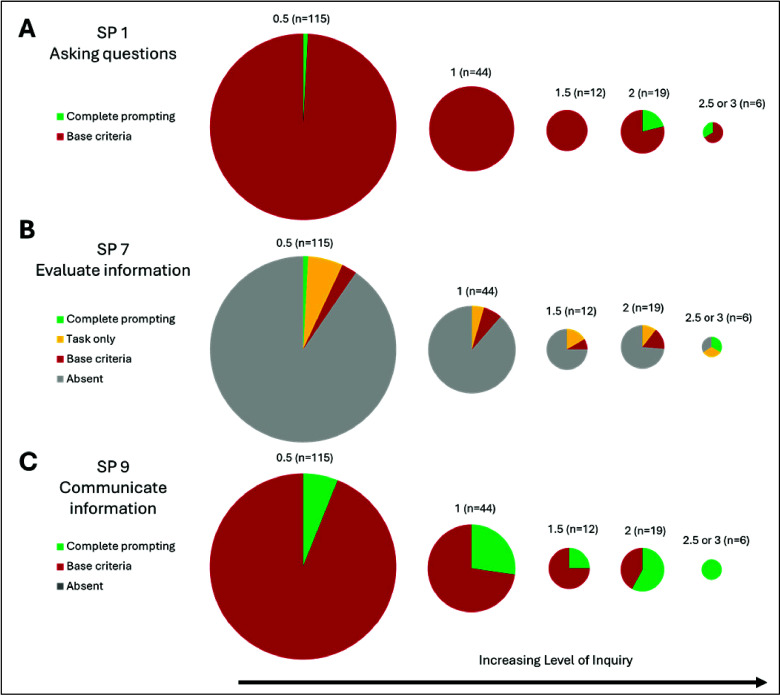
Percentage of laboratory materials that provide prompted opportunities for engaging in a given science practice separated by their level of inquiry. The size of the circle corresponds to the number of laboratory experiments within each inquiry level. (A) Results for *asking questions* (SP1), (B) *evaluating information* (SP7), and (C) *communicating information* (SP9).

For three science practices, there was a general increase in prompted opportunities to engage students in some science practices as the level of inquiry increased. This pattern was observed for *asking questions* (SP1), *evaluating information* (SP7), and *communicating information* (SP9) as illustrated in Figure 1.

For these three science practices, the higher level of inquiry laboratory experiment provided less structure and required students to generate these components for the experiment. At the highest level of inquiry, authentic inquiry (level 3), a student must generate a research question, so it is unsurprising that we rarely see prompted opportunities for students to engage in *asking questions* (SP1) until that level. For *evaluating information* (SP7), the 3D-LAP defines this practice as having students evaluate an outside video, article, student solution, or excerpt from a conversation that makes one or more assertions. In higher levels of inquiry laboratory experiments, such as independent or group projects, students often do a literature search or find external information they must evaluate, in contrast to the lower-level materials where they are often provided the necessary background/theory for the laboratory experiment. For *communicating information* (SP9), we observed that instructors often had students report their results in an Excel sheet or through post-laboratory questions for lower level of inquiry laboratory materials, and it was not until the higher level of inquiry laboratory experiments, often project-based experiments, where students were asked to write up a formal laboratory report or create a presentation. For all three of these science practices, as students engage with laboratory experiences that are more authentic to that of scientists and thus farther into the community of practice, students typically encounter more complete opportunities to engage in *asking questions*, *evaluating information*, and *communicating information*.

**Figure 2:**
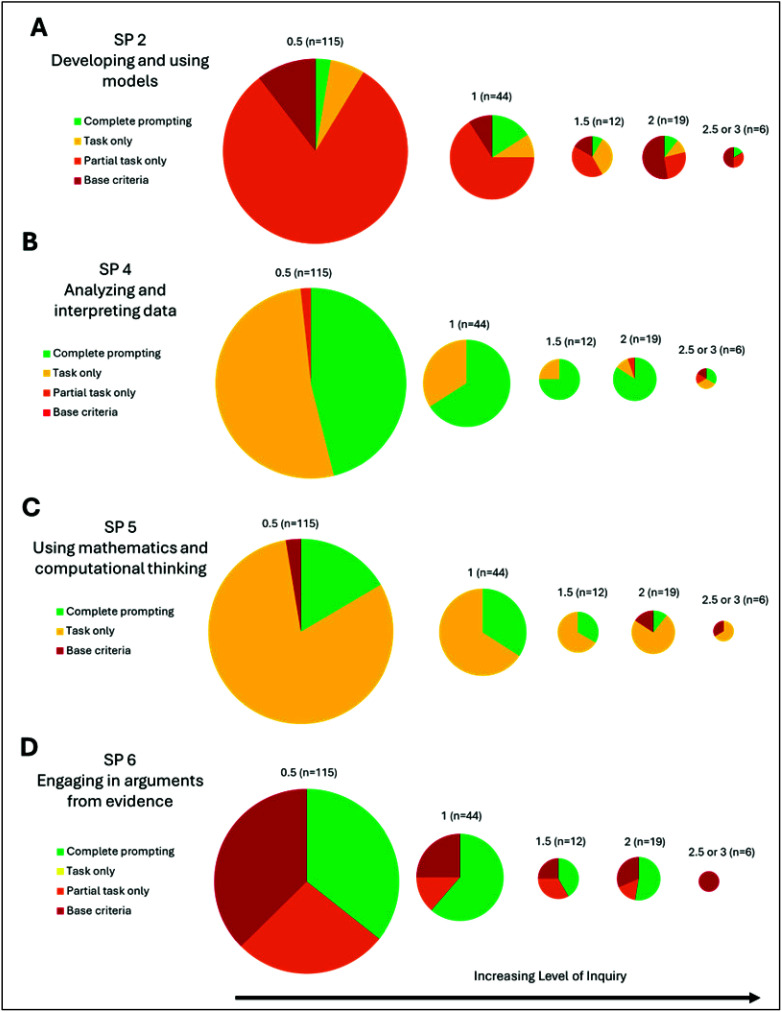
Percentage of laboratory materials that provide prompted opportunities for engaging in a given science practice, separated by their level of inquiry. The size of the circle corresponds to the number of laboratory experiments within each inquiry level. Results for (A) *developing and using models* (SP2), (B) *analyzing and interpreting data* (SP4), (C) *using mathematics and computational thinking* (SP5), and (D) *engaging in arguments from evidence* (SP6).

There was an initial increase in prompted opportunities to engage in a given science practice with increasing levels of inquiry followed by a decrease at the highest levels of inquiry for four science practices: *developing and using models* (SP2), *analyzing and interpreting data* (SP4), *using mathematics and computational thinking* (SP5), and *engaging in arguments from evidence* (SP6) as seen in Figure 2.

When we consider what students must do when they are engaged in laboratory experiments that are higher in the level of inquiry, we would anticipate that they will engage in many of these science practices, so it initially seemed surprising to see a decrease in prompted opportunities with the highest levels of inquiry. However, experiments with higher levels of inquiry often decrease the scaffolding or support that students are explicitly provided with in their laboratory handouts. Since the 3D-LAP captures the explicit prompting for the opportunity to engage in a science practice within a laboratory experiment, if the scaffolding that prompts students to engage in a practice is removed, the results from the 3D-LAP will reflect that. One example is *engaging in arguments from evidence* (SP6). We often observed opportunities to engage in *engaging in arguments from evidence* (SP6) in post-laboratory questions. In laboratory experiments of higher levels of inquiry there were often no post-laboratory questions to scaffold student thinking about their data. This does not mean that students will not engage in that practice but rather that opportunities to engage in the practice are not prompted by the laboratory materials. Considering the lens of situated learning, we would anticipate that, as a student moves from the periphery further into the community, they would develop proficiency with science practices such that scaffolding could be removed and students would engage in the science practice without prompting. Further work investigating evidence of student engagement of science practices within student coursework and laboratory observations as well as student and faculty interviews about norms of the course would help to illuminate the practices in which students actually engage in these more authentic laboratory experiences.

**Figure 3:**
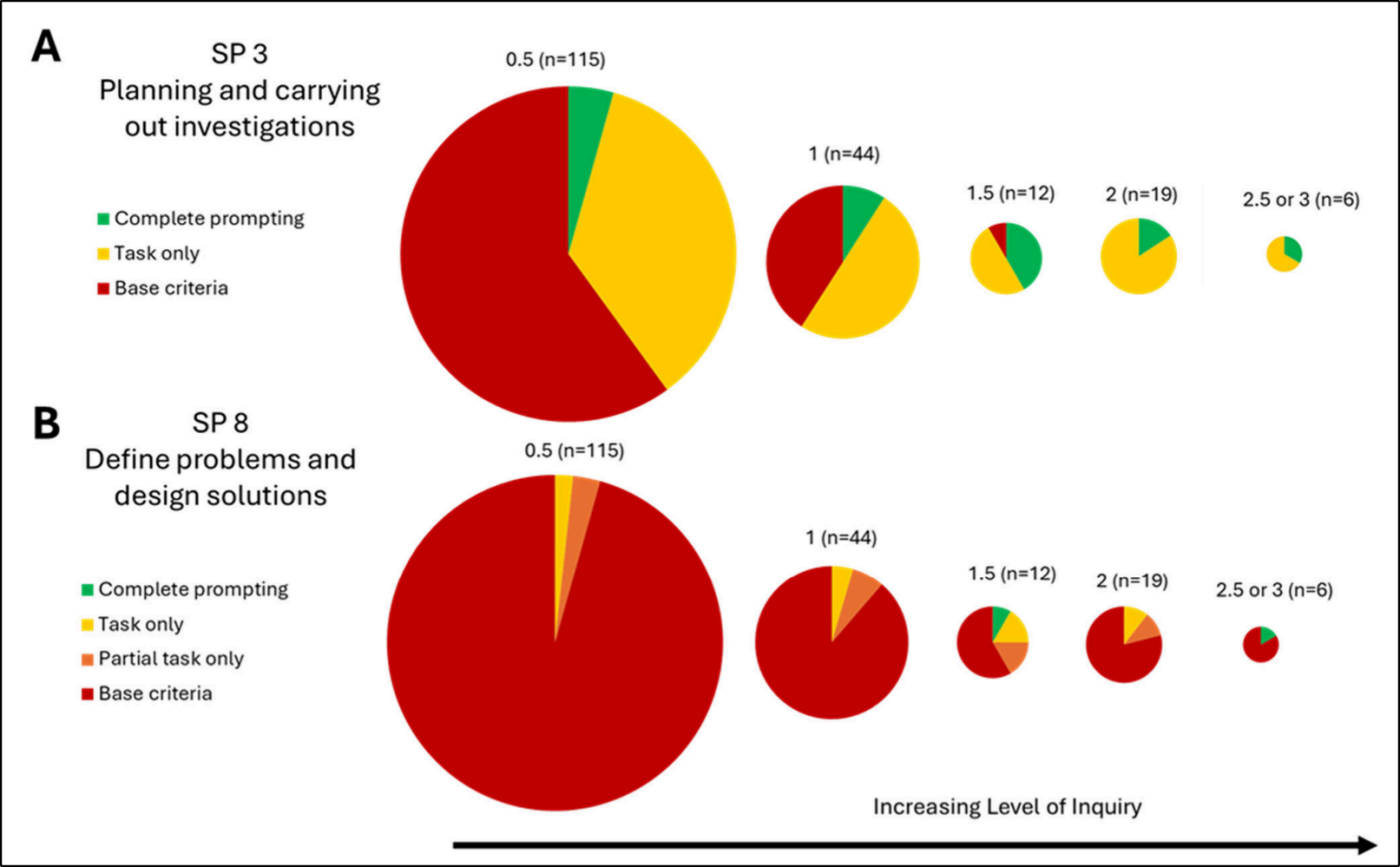
Percentage of laboratory materials that provide explicit opportunities for engaging in a given science practice separated by their level of inquiry. The size of the circle corresponds to the number of laboratory experiments within each inquiry level. (A) Results for *planning and carrying out investigations* (SP3) and (B) *defining problems and designing solutions* (SP8).

There were two science practices that did not fall into either of the previous patterns. One was *planning and carrying out investigations* (SP3), and the other was the engineering practice of *defining problems and designing solutions* (SP8), as seen in Figure 3.

For *planning and carrying out investigations* (SP3) we see an increase through level 1.5, a dip for levels 2 and 2.5, and finally an increase with level 3. When both criteria 1 and 2 were present, as indicated by “task only”, the research question for the investigation has been established (criterion 1) and the student must either (1) “describe or design an investigation or (2) identify the observations required to answer the question or test the claim or hypothesis” (criterion 2). The third criterion that completes the opportunity to engage in *planning and carrying out investigations* requires the student to “justify how their description, design, or observations can be used to answer the question or test the claim or hypothesis”. In levels 2 and 2.5 we see that there is minimal “fully met” and majority “task only”, indicating that this last criterion where students must justify their description, design, or observations is absent. So, while students are making experimental decisions in these laboratory activities, they were often not prompted by the laboratory materials to justify those decisions. Ideally students have a rationale for making their experimental design decisions, but without the prompting to provide a justification, students are not being assessed on that aspect of *planning and carrying out investigations*. It is possible the instructor may verbally probe students for their reasoning or justification during the laboratory work, but more explicit prompting in laboratory handouts or report directions would enhance the opportunity for students to display proficiency.

For the engineering practice of *defining problems and designing solutions* (SP8), we see that this practice was not emphasized in our data set by the high percentage of “base criteria”, indicating that only the base criteria, defining the research question or phenomenon to be studied (criterion 1), were present. These base criteria alone are not enough to say that any extent of this practice is prompted. In the instances where more than the base criterion was present, students were asked to either “identify findings from their investigation that will be used in designing a solution to a problem” (criterion 2), “design or build a tangible product from the results of their investigation” (criterion 3), or “discuss how they weighed or prioritized competing criteria in designing a solution” (criterion 4). There were instances where some of these criteria would appear but not necessarily in order, unlike the other science practices that had a more hierarchical structure. This engineering practice was most commonly observed in the MICRO Make Your Own Microfluidic Device laboratory experiment or other laboratory experiments that required students to engage in iterative design planning or device fabrication.

### Component of Laboratory Material vs Opportunities to Engage in Science Practices

In analyzing the laboratory experiments, we noticed that prompted opportunities to engage in certain science practices tended to appear in different parts of the laboratory materials. As our data has indicated that intentional changes to the level of inquiry may not result in an increase in opportunities to engage in science practices, investigating the ways that science practices were prompted has direct implications for intentionally designing laboratory experiences that help cultivate proficiency with science practices. The percentage of laboratory materials that explicitly prompt for a given science practice within a given laboratory component (pre-lab questions, background, procedure, data analysis, and post-lab questions) is summarized in Table 6.

**Table 6:**
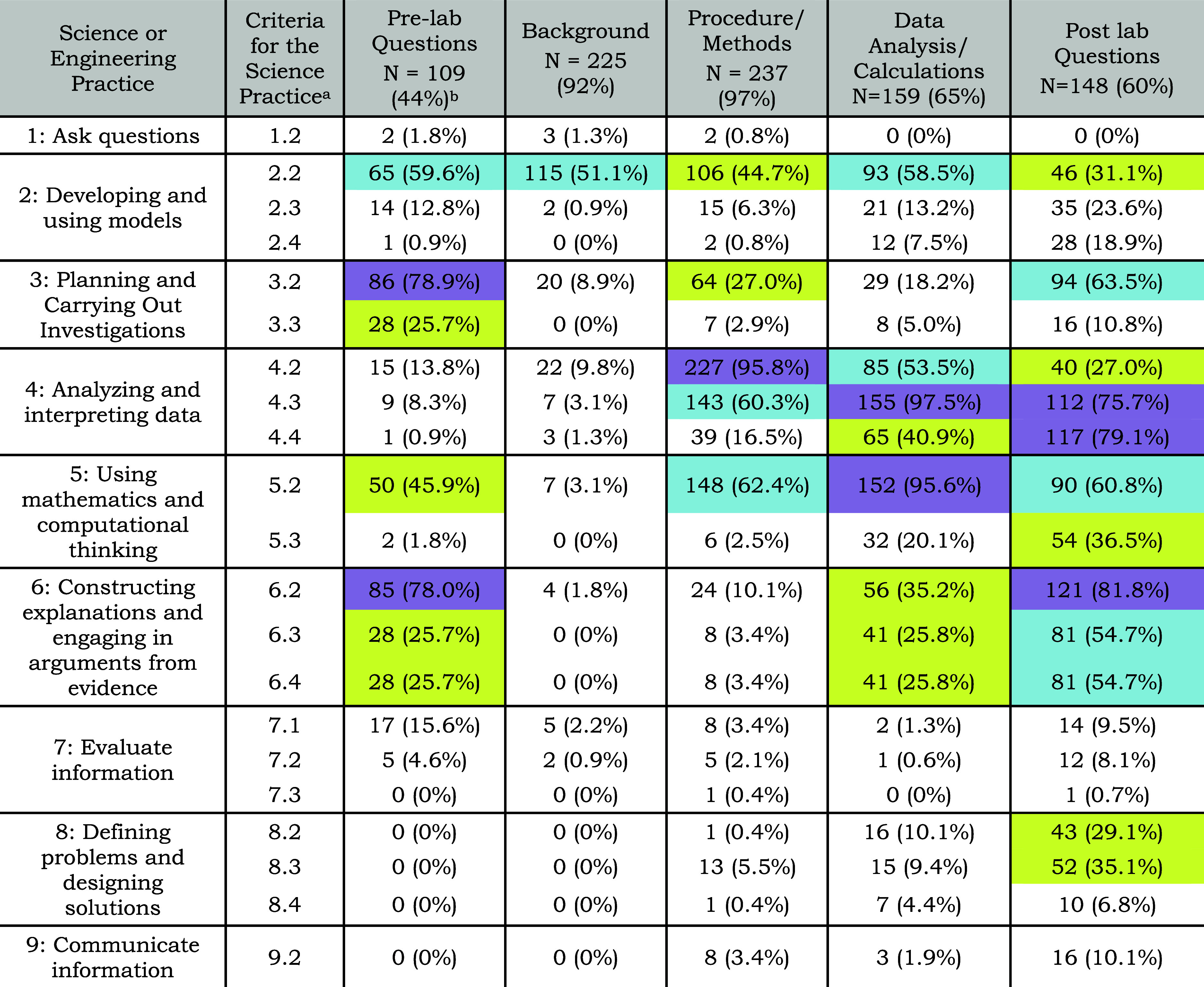
Percentage of Laboratory Components That Contained Prompted Opportunities to Engage Students in Science Practices

a Definitions of the different criteria of each science practice can be referenced in the codebook provided in the Supporting Information. The first criterion is omitted for all the science practices as it is establishing a research question to be investigated or a phenomenon of interest to study, which was always present. Criterion 7.1 is included as it is different from the rest of the science practice’s first criteria and was not always present.

b Percentages of 75% or greater are highlighted purple, percentages of 50% or higher are highlighted blue, and percentages of 25% or higher are highlighted yellow.

Looking across all laboratory experiments, most laboratory materials contained the laboratory component of background (92%) and procedure (97%), but not all laboratory materials contained pre-laboratory questions (44%), data analysis (65%), or post-laboratory questions (60%). This reflects the variations in the structure of the laboratory materials used by the different participants. Trends for where in the laboratory materials each science practice appeared are discussed below along with suggestions for ways to prompt for criteria not seen in our data set.

#### Asking Questions, Evaluating Information, Communicating Information

Since our data set did not contain many laboratory experiments that provided prompted opportunities to engage in *asking questions* (SP 1), *evaluating information* (SP 7), or *communicating information* (SP 9), we did not see high percentages of overlap with any laboratory component for those science practices. As seen in Table 6, when opportunities to engage in *asking questions* (SP 1) were present, it was in the pre-laboratory questions/assignment, background, or procedure. These prompts were present in the highest levels of inquiry experiments, most often laboratory projects, where students were required to generate a research question. A potential way that instructors could use post-lab questions to prompt students to engage in *asking questions* (SP1) is by having them write a research question that emerges from the laboratory experiment they just completed.

When opportunities to engage in *evaluating information* (SP 7) were present, it was in pre-laboratory questions/activities or post-laboratory questions. These types of prompts quite often had students search for resources or information on their own and then evaluate them. Students were directed in these questions to use the literature to learn more about theory, a technique they would use in the laboratory experiment, or to learn more about the phenomenon that was the focus of the laboratory experiment. The other type of pre-laboratory activity that engaged students in *evaluating information* was when students had to search literature for a method or procedure, often in a higher-inquiry, project-based laboratory experiment.

When opportunities to engage in *communicating information* (SP 9) were present, it was commonly in the post-laboratory questions/assignment. These activities prompted students to write a formal laboratory report, prepare a scientific presentation to other scientists, or translate their findings to a general audience. There was not a high percentage of this science practice as many faculty had their students answer post-laboratory questions rather than create a communication product.

#### Developing and Using Models

The most prevalent criterion present for *developing and using models* (SP 2) was criterion 2.2, where students were either given a model or prompted to create a model. This criterion appeared across all the laboratory components as models were quite often provided in the form of necessary chemical equations, mathematical equations, laboratory equipment setup, or representations of chemical phenomena. Students were also often prompted to generate models in their pre-laboratory questions, data analysis/calculations, and post-laboratory questions. We saw less of criteria 2.3 and 2.4, which require students to use the model and to link meaning to the representation. As seen in Table 6, post-laboratory questions sometimes prompted students to give an interpretation or explanation of a model.

#### Planning and Carrying out Investigations

The most common criterion for *planning and carrying out investigations* (SP 3) was 3.2, which required students to describe or design an investigation. This criterion was typically observed in the pre-laboratory questions/activities as students were tasked with planning out part or all of the laboratory experiment prior to conducting the experiment. We also observed pre-laboratory and post-laboratory questions where students had to describe part or all of the investigation. Criterion 3.3 occurred less frequently as it required students to provide justification for the laboratory design. When this criterion did appear, it was quite often in the pre-laboratory questions or post-laboratory questions, as they prompted for a written explanation. Encouraging students to justify their proposed methods can help to highlight gaps in their understanding surrounding the research question they are trying to answer and selecting appropriate methods for answering that question.

#### Analyzing and Interpreting Data

Many of the criteria for *analyzing and interpreting data* (SP 4) were observed across the different laboratory components. For the first criterion (4.2) students were given or had to generate a representation of the data, which was most often seen in the procedure where students were collecting the data or the data analysis/calculations where students were working up the data. Criterion 4.3 required students to analyze the data or gave the students an analysis of the data, which was most frequently in the data analysis/calculations section as well as the procedure and post-laboratory questions. These sections prompted students to analyze the data in a specified manner, often generating a graph, using an equation/test, or looking for a trend in the data. The last criterion (4.4) required the student to interpret the results in the context of the scientific question that the laboratory experiment is looking to answer. This was most frequently prompted for in the post-laboratory questions where students had to provide an explanation or interpretation of their results. Adding prompts to consider the interpretation of results during the procedure could help students with laboratory experiments where they have to iterate on an experiment, such as a higher inquiry laboratory experiment, and to encourage the habit of critically thinking about data as it is being actively collected.

#### Using Mathematics and Computational Thinking

For *using mathematics and computational thinking* (SP 5), criterion 5.2 was seen most frequently, which required students to perform a calculation, generate a mathematical representation, or determine a relationship between different parameters. This was most observed in the data analysis/calculations portion of a laboratory experiment but was also seen in the pre-laboratory questions, procedure, and post-laboratory questions. Similar to criterion 4.3, these prompts had students analyzing data in a specified mathematical matter, quite often generating graphs or a mathematical representation. When criterion 5.3 was observed, it was often in the post-laboratory questions where students had to provide a consequence or interpretation of their calculation, mathematical representation, or analysis. This may be a helpful exercise for the pre-laboratory questions and background section to help students make sense of the mathematical models that they will be using throughout the experiment.

#### Engaging in Arguments from Evidence

The most common criterion present for *engaging in arguments from evidence* (SP 6) was 6.2, which either provided students with a claim or had them generate a claim based on a given event, observation, or phenomenon. The majority of the time students were prompted to generate a claim, which was most often seen in pre-laboratory and post-laboratory questions that required students to write a direct claim to answer a question. When criteria 6.3 and 6.4 were present, requiring the student to provide evidence or scientific principles (6.3) and then connect them to the claim (6.4), they were frequently in post-laboratory questions that prompted students to explain their findings in the context of the research goal of the experiment.

#### Defining Problems and Designing Solutions

For the engineering practice *defining problems and designing solutions* (SP 8), the most common criteria present were 8.2 and 8.3. Criterion 8.2 had students identify findings from their investigation that can be used in designing a solution and how they will be used. Criterion 8.3 had students design or build a tangible product from the results of their investigation. When either of these criteria were present, it was most often in the post-laboratory questions due to the nature of this science practice to use results from one investigation to design a solution for another. While the engineering practice of *defining and designing solutions* was not prevalent in our data, one example was with the MICRO Make Your Own Microfluidic Device laboratory experiment where students were asked to improve the design of a microfluidic device based on the results of their laboratory experiment.

### Participants’ Modified MICRO Laboratory Experiments Compared to the Original MICRO Laboratory Experiments

As part of the data collection process, participants were asked to submit all of the laboratory materials they used during the semester of implementation, including the MICRO laboratory experiments they implemented. Since faculty were encouraged to make modifications to the MICRO laboratory materials to fit their specific institution and course curriculum, we examined the nature of modifications that faculty made and if those modifications impacted the prompted opportunities for students to engage in science practices. Table 7 summarizes the number of participants who implemented each MICRO laboratory experiment, the number of participants who used the laboratory without modifications, and the number of participants who made modifications to the laboratory materials. Two laboratory experiments were excluded from this analysis, as the instructors did not submit sufficient information to be able to compare to the original MICRO laboratory experiment.

**Table 7:** MICRO Laboratory Experiments Participants Used during Their Semester of Implementation

MICRO Laboratory Experiment	Number of Participants Who Used It without Modification	Number of Participants Who Used It with Modifications	Total
Vitamin C	1	3	4
Cream of Tarter/Baking Soda Titration	3	3	6
Copper Electrochemistry	2	2	4
Milk Protein	3	6	9
Make Your Own Microfluidic Device	4	7	11
Vinegar Titration	5	7	12

It can be observed that, for all of the MICRO laboratory experiments implemented by participants, 50% or more made modifications to the laboratory materials. These modifications ranged in scope and nature. Some small modifications such as the removal of a learning objective or a small change in the procedure such as a substituted reagent did not result in changes in the opportunities to engage in science practices. However, we noticed some modifications, such as the addition or the elimination of laboratory components, that resulted in differences in the prompted opportunities to engage students in science practices when compared with the original MICRO laboratory materials. Here we outline the different types of modifications that we observed and their impact on science practice opportunities.

The first type of modification was the addition of laboratory components that resulted in more prompted opportunities to engage students in some of the science practices. A few participants chose to add a more formal assessment to the MICRO laboratory experiments they implemented in addition to the post-laboratory questions already provided. Some faculty had students write a formal report, often following the guidelines of a journal such as the *American Chemical Society*. Other faculty had students create a poster presentation, many of which also required their students to present at their institution’s research and creativity symposium. In both instances, the criteria for *communicating information* (SP9) were fully met when they previously were not. A few other participants added an assessment where students had to generate a video to report their findings. For these, in addition to *communicating information* (SP9) we saw increases in the opportunities to engage in *using mathematical and computational thinking* (SP5) and *engaging in arguments from evidence* (SP 6) as students were prompted to explain their results in the context of the research question or phenomenon being studied.

Another significant type of modification was the elimination of laboratory components from the experiments. Often faculty removed pre-laboratory or post-laboratory questions from the MICRO laboratory experiments. When participants removed pre-laboratory questions, we often observed fewer prompted opportunities to engage in *developing and using models* (SP2), *planning and carrying out investigations* (SP3), *using mathematics and computational thinking* (SP5), and *engaging in arguments from evidence* (SP 6). The pre-laboratory questions in the MICRO laboratory experiments were intentionally designed to get students involved in planning elements prior to completing the laboratory experiment or thinking about the nature of the data or data analysis they would do for the laboratory experiment. While some faculty just removed a question or two, other faculty removed the pre-laboratory questions all together. While they may have prepared students in other ways not captured in our material analysis, removing pre-laboratory activities can be detrimental as pre-laboratory activities help scaffold the complex nature of laboratory work.^48,49^ The other scenario that occurred was the removal of post-laboratory questions. As with the pre-laboratory questions, some faculty removed one or two post-laboratory questions while others removed the whole section or replaced it with an alternate assignment. One participant replaced the post-laboratory questions with an Excel template where students plugged in their data, and it computed their results. With the removal of post-laboratory questions, we saw reductions in the opportunities to engage in *analyzing and interpreting data* (SP4), *engaging in arguments from evidence* (SP6), and the engineering practice of *defining problems and designing solutions* (SP8). The post-laboratory questions were intentionally designed to have students explain and argue their results, often the last criterion of these science practices, so removing the post-laboratory questions removed the explicit prompting for students to do this important reflection and sense-making of their results. Through the lens of constructive alignment, if engagement in these science practices is a learning goal, the activities and assessments students complete must include opportunities to engage in these science practices to develop proficiency. Removing these prompts from the laboratory tasks removes opportunities for students to demonstrate proficiency with science practices as learning goals.

## LIMITATIONS

There are a few limitations of this study. One limitation is that the course materials analyzed for this study were all from analytical chemistry, mainly the course often referred to as “Quantitative Analysis”, so study findings are not representative of all chemistry courses. Additionally, participants in the MICRO project applied to be a part of the project, so the findings may not be representative of the laboratory materials of all analytical chemistry faculty. Another limitation was that the number of laboratory experiments that were considered higher-inquiry laboratory experiments was small in number, limiting the claims that could be made. For this study we only collected the laboratory materials that faculty used in their courses along with survey and interview data solely targeting faculty’s beliefs. If we had collected other types of data such as student backgrounds and the overall course structure and management we might have been able to develop a more wholistic description of the courses analyzed.^49^ Finally, the analytical tool used to analyze the laboratory materials, the 3D-LAP, only characterized explicit prompting of opportunities to engage students in science practices. If norms were established in other manners, such as in-class discussions or verbal instruction, these opportunities were not identified. Additionally, the level of student engagement in the science practices or students’ level of proficiency cannot be determined with this tool. It stands to reason that, even if there is an opportunity in the laboratory materials for a student to engage in a given science practice, they may not actually engage in the science practice for a variety of reasons. More work should be done to capture the enacted curriculum, rather than intended, as well as students’ actual engagement and proficiency with the science practices.

## IMPLICATIONS

### Implications for Materials Design

There are several implications from our study findings for chemistry curriculum design and, in particular, laboratory curricula. In our data we saw limited opportunities for students to engage in *asking questions* (SP 1), *evaluating information* (SP 7), and *communicating information* (SP 9). These could be science practices that may not be commonly emphasized in analytical chemistry curricula and may be an area to enhance laboratory materials to give students more practice with those science practices. An example of how *asking questions* (SP 1) could be prompted is to have students generate a new research question as part of the post-laboratory questions/activity inspired by the work they completed in the experiment.

In our data, we saw that many laboratory materials were close to providing “full” opportunities to have students engage in science practices except they lacked the last criteria, generally the interpretation/justification piece. This interpretation/justification piece is important as it can reveal a student’s level of proficiency with a given science practice. When we did see this criterion, it was often in the pre- and post-laboratory questions. Instructors or curriculum designers could consider adding prompts such as pre- and post-laboratory questions/activities to materials to prompt for these interpretation/justification pieces, so students have more complete opportunities to engage in these science practices.

Another implication of this work for material design can be drawn from the finding that, as the laboratory materials were enhanced to engage students in higher levels of inquiry, the materials did not inherently have more prompted opportunities to engage students in many of the science practices. In addition to evaluating laboratory materials for their level of inquiry, evaluating laboratory materials for their prompted opportunities to engage students in science practices is important to highlight alignment or misalignment with promoting scientific practice. When designing a higher level of inquiry laboratory materials, it is important to consider what explicit prompting to engage in a science practice may have been removed and how students will be assessed on that practice.

Another implication of this work is the role of different laboratory components and how they can prompt students to engage in different science practices. We saw that, for many of the science practices, the interpretation/justification criteria of the practices were commonly prompted for in the pre- and post-laboratory questions, allowing students to fully demonstrate their proficiency with a given practice. Removing these sections would remove these prompts to engage in science practices. In fact, we saw that, in the modifications that faculty made to the MICRO laboratory materials to eliminate pre- and post-laboratory questions, some of the opportunities to engage in certain science practices were reduced. We also saw the alternative: as post-laboratory questions or laboratory report assignments were added, we saw increases in prompted opportunities to engage in some of the science practices. Careful consideration of what science practice goals you have for students in a laboratory experiment and where in the laboratory experiment or materials they are prompted to demonstrate those practices is needed to design aligned laboratory materials.

### Implications for Research

In previous studies applying the 3D-LAP to course materials, authors have attributed opportunities to engage in a science practice if all of the criteria for a given science practice were present. We took an approach similar to Raycroft and Flynn where they tracked which criteria were present for each science practice to determine the extent to which laboratory materials gave students the opportunity to engage in a science practice.^50^ This approach allowed us to see that many laboratory materials were short of a “complete prompting” opportunity to engage students in a science practice and could easily be enhanced by prompting students to provide a justification or interpretation. This type of approach allows for a more in-depth analysis and can provide tangible ways to improve course materials to align with the desired science practices. Through the lens of situated learning, it could be interesting to examine the types of opportunities that students have to engage in each science practice as they move from the periphery in introductory chemistry courses to courses at the end of their chemistry degree to see if the types of opportunities move toward more “complete prompting” opportunities as they develop proficiency with the science practices.

We observed in our data that shifts to higher levels of inquiry of laboratory materials did not always result in more prompted opportunities to engage students in certain science practices. We hypothesized that, with the reduction in scaffolding accompanying the higher levels of inquiry, the explicit prompting to engage in these science practices was also removed. It is possible that, in place of these written scaffolds and prompts, the course instructors or classroom norms had students engaging in these science practices, but they were not identified due to limits of the 3D-LAP. Future research could investigate the role of instructors in prompting and supporting student engagement in science practices as well as established classroom norms or activities. It is also necessary to investigate students’ level of proficiency with the science practices when they have prompted opportunities to engage in a science practice and when they are not prompted.

The MICRO workshops focused primarily on training faculty with inquiry-based laboratory experiments and did not explicitly focus on creating prompts to engage students in science practices. This may be one of the reasons that we did not observe shifts in the opportunities to engage in science practices but did observe shifts for levels of inquiry. It would be worthwhile to investigate the impact of a workshop primarily focused on embedding prompts to engage in science practices in laboratory experiments. Additionally, studies collecting additional data sources, such as information about course management and organization and course curriculum, may provide a more complete picture of the alignment of course materials with the science practices.

## Conclusion

The MICRO project was designed to support analytical chemistry faculty in adopting inquiry-based instruction. Analysis of their laboratory materials for level of inquiry, reported previously,^45^ and for opportunities to engage in science practices using a modified version of the 3D-LAP lead to some conclusions and insight for laboratory material design. The analysis of laboratory materials used by participants prior to and during their semester of involvement in the MICRO project revealed that changes to the laboratory materials to increase the level of inquiry did not necessarily result in increased opportunities provided to engage students in science practices as previous research had indicated. From our data, we observed two patterns of prompted opportunities to engage in a science practice with a level of inquiry. For some of the science practices, a higher level of inquiry resulted in more prompted opportunities to engage in science practice. However, for other science practices, a higher level of inquiry resulted in fewer prompted opportunities to engage in those science practices. Our data suggests that the reduced scaffolding associated with the higher levels of inquiry could explain the decrease in prompting for students to engage in those science practices. Overall, our data suggest that increasing opportunities to engage in science practices may need more intention than increasing the level of the laboratory experiment. This points to the need for training on science practices in conjunction with professional development efforts for increasing the level of inquiry.

We also observed that different science practices were more commonly prompted for in different components of laboratory experiments such as pre-laboratory and post-laboratory questions. In particular, the explanatory parts of many of the science practices were most often prompted in the pre-laboratory and post-laboratory questions/activities. With this, additions or eliminations to laboratory components of the MICRO laboratory experiments that faculty made often impacted the extent to which opportunities were provided to engage students in certain science practices. This reveals the significance of these laboratory components in providing prompts to have students display proficiency in science practices.

## Supporting Information Available

Complete codebook for identifying opportunities to engage in science practices (PDF, DOCX)
Additional studies investigating differences in opportunities to engage in science practices between pre- and post-laboratory materials (PDF, DOCX)
