# Supplementary material for: Bridging the Science Practices Gap: Analyzing Laboratory Materials for Their Opportunities for Engagement in Science Practices
Source: J Chem Educ. 2025 Feb 11;102(3):970–83. doi: 10.1021/acs.jchemed.4c00744 (PMC11905284; doi:10.1021/acs.jchemed.4c00744)
Supplement: Supplementary file 1 — ed4c00744_si_001.pdf [file ed4c00744_si_001.pdf]

Supporting Information

Bridging the science practices gap: Analyzing laboratory materials for their opportunities for engagement in science practices

Andrea L. Van Wyk[1^], Ardith Bhinu[1], Kimberley A. Frederick[2], Marya Lieberman[3], Renée S. Cole[1]

[1]Department of Chemistry, University of Iowa, Iowa City, IA 52242, USA
[2]Department of Chemistry, Skidmore College, Saratoga Springs, NY 12866, USA
[3]Department of Chemistry and Biochemistry, University of Notre Dame, Notre Dame, IN 46556, USA
^Present Address: Department of Chemistry and Physics, Drake University, Des Moines, IA 50311, USA

Codebook for opportunities for engagement in science practices in chemistry laboratory experiments

1. Unit of Analysis

The unit of analysis is one student handout of a laboratory experiment. The specific part(s) in the student handout of the laboratory experiment can vary from science practice to science practice. Oftentimes pre-laboratory questions or post-laboratory questions/assignments provide places for students to engage in some of the science practices.

2. Science Practices

*The following definitions and criteria are modified from the Three-Dimensional Learning Assessment Protocol (3D-LAP) by Laverty et. al. and extra criteria put forth in Carmel et. al. 2019 (SP 8 and 9 below). Deviations from the two publications will be indicated.

SP1: Ask Questions

Criteria 1: Laboratory experiment gives an event, observation, phenomenon, data, scenario, or model.

Criteria 2: Laboratory experiment asks students to generate an empirically testable question about the given event, observation, phenomenon, data, scenario, or model.

SP2: Developing and Using Models

Criteria 1: Laboratory experiment gives an event, observation, phenomenon for the student to explain or make a prediction about.

Criteria 2: Laboratory experiment gives a representation/model or asks student to construct a representation/model

Criteria 2a: Laboratory experiment gives a representation/model

Criteria 2b: Laboratory experiment asks student to construct a representation/model

Criteria 3: Laboratory experiment asks student to explain or make a prediction using the representation about the event, observation, or phenomenon.

Criteria 4: Laboratory experiment asks student to provide the reasoning that links the representation to their explanation or prediction.

SP3: Planning and Carrying Out Investigations

Criteria 1: Laboratory experiment poses a scientific question, claim, or hypothesis to be investigated.

Criteria 2: Laboratory experiment asks student to describe or design an investigation, or identify the observations required to answer the question or test the claim or hypothesis.

Criteria 3: Laboratory experiment asks student to justify how their description, design or observations can be used to answer the question or test the claim or hypothesis.

SP4: Analyzing and Interpreting Data

Criteria 1: Laboratory experiment gives a scientific question, claim or hypothesis to be investigated.

Criteria 2: Laboratory experiment gives a representation of the data (e.g., table or graph, or lists of observations) provided to answer the question or test the claim or hypothesis.

> Criteria 2a: Laboratory experiment gives a representation of the data (e.g., table or graph, or lists of observations) provided to answer the question or test the claim or hypothesis.
>
> Criteria 2b: Laboratory experiment has students generate a representation of the data (e.g., table or graph, or lists of observations) provided to answer the question or test the claim or hypothesis.

Criteria 3: Laboratory experiment gives an analysis of the data or asks student to analyze the data moving towards the goal of answering the research question/goal.

> Criteria 3a: Laboratory experiment gives an analysis of the data
>
> Criteria 3b: Laboratory experiment asks student to analyze the data

Criteria 4: Laboratory experiment asks student to interpret the results or assess the validity of the conclusions in the context of the scientific question, claim, or hypothesis.

SP5: Using Mathematical and Computational Thinking

Criteria 1: Laboratory experiment gives an event, observation, or phenomenon.

Criteria 2: Laboratory experiment asks student to perform a calculation or statistical test, generate a mathematical representation, or demonstrate a relationship between parameters in order to obtain the correct answer.

Criteria 3: Laboratory experiment asks student to give a consequence or an interpretation (not a restatement) in words, diagrams, symbols, or graphs of their results in the context of the given event, observation or phenomenon.

SP6: Constructing Explanations and Engaging in Arguments from Evidence

Criteria 1: Laboratory experiment gives an event, observation, or phenomenon.

Criteria 2: Laboratory experiment gives or asks student to make a claim based on the given event, observation, or phenomenon.

> Criteria 2a: Laboratory experiment gives a claim based on the given event, observation, or phenomenon.
>
> Criteria 2b: Laboratory experiment asks students to make a claim based on the given event, observation, or phenomenon.

Criteria 3: Laboratory experiment asks students to provide scientific principles or evidence in the form of data or observations to support the claim.

Criteria 4: Laboratory experiment asks student to provide reasoning about why the scientific principles or evidence support the claim.

SP7: Evaluate Information

Criteria 1: Laboratory experiment gives, or student obtains, an excerpt from a conversation, article, student solution, or video that makes one or more assertions.

Criteria 2: Laboratory experiment gives a conclusion about the validity of the assertion(s) or asks student to make a conclusion about the validity of the assertion(s) or reconciliation of multiple assertions.

> Criteria 2a: Laboratory experiment gives a conclusion about the validity of the assertion(s) or reconciliation of multiple assertions
>
> Criteria 2b: Laboratory experiment asks student to make a conclusion about the validity of the assertion(s) or reconciliation of multiple assertions

Criteria 3: Laboratory experiment asks student to provide reasoning to support their conclusion(s) about the validity of the assertion(s) or reconciliation with data, observations, or scientific principles.

SP8: Defining Problems and Designing Solutions

Students are asked to design/build something to serve as a function as a result of their investigation.

Criteria 1: Laboratory experiment gives an event, observation, or phenomenon, scenario, or model.

Criteria 2: Laboratory experiment asks students to identify the findings from their investigation that will be used in designing the solution and how they will be used.

Criteria 3: Laboratory experiment asks students to design or build a tangible product from the results of their investigation.

Criteria 4: Laboratory experiment asks students to discuss how they weighed or prioritized competing criteria, such as function, feasibility in production of design, and/or, in designing a solution.

SP9: Communicate Information

Students are asked to present their findings of their experiments to an audience.

Criteria 1: Laboratory experiment gives an event, observation, phenomenon, scenario, or model.

Criteria 2: Laboratory experiment asks students to communicate their findings to an audience beyond the instructor, either another expert in chemistry or a general public audience.*

> Criteria 2a: Laboratory experiment asks students to communicate their findings to another scientist.*
>
> Criteria 2b: Laboratory experiment asks students to communicate their findings to a general public audience.

*This is a deviation from Carmel et. al. who previously just specified a general audience.
