# Supplementary material for: Bridging the Science Practices Gap: Analyzing Laboratory Materials for Their Opportunities for Engagement in Science Practices
Source: J Chem Educ. 2025 Feb 11;102(3):970–83. doi: 10.1021/acs.jchemed.4c00744 (PMC11905284; doi:10.1021/acs.jchemed.4c00744)
Supplement: Supplementary file 2 — ed4c00744_si_002.docx [file ed4c00744_si_002.docx]

**Supporting Information**

**Bridging the science practices gap: Analyzing laboratory materials for their opportunities for engagement in science practices**

Andrea L. Van Wyk^1^^, Ardith Bhinu^1^, Kimberley A. Frederick^2^, Marya Lieberman^3^, Renée S. Cole^1^

^1^Department of Chemistry, University of Iowa, Iowa City, IA 52242, USA
^2^Department of Chemistry, Skidmore College, Saratoga Springs, NY 12866, USA

^3^Department of Chemistry and Biochemistry, University of Notre Dame, Notre Dame, IN 46556, USA

^Present Address: Department of Chemistry and Physics, Drake University, Des Moines, IA 50311, USA

**Codebook for opportunities for engagement in science practices in chemistry laboratory experiments**

# Unit of Analysis

The unit of analysis is one student handout of a laboratory experiment. The specific part(s) in the student handout of the laboratory experiment can vary from science practice to science practice. Oftentimes pre-laboratory questions or post-laboratory questions/assignments provide places for students to engage in some of the science practices.

# Science Practices

*The following definitions and criteria are modified from the Three-Dimensional Learning Assessment Protocol (3D-LAP) by Laverty et. al. and extra criteria put forth in Carmel et. al. 2019 (SP 8 and 9 below). Deviations from the two publications will be indicated.

## **SP1: Ask Questions**

Criteria 1: Laboratory experiment gives an event, observation, phenomenon, data, scenario, or model.

Criteria 2: Laboratory experiment asks students to generate an empirically testable question about the given event, observation, phenomenon, data, scenario, or model.

## **SP2: Developing and Using Models**

Criteria 1: Laboratory experiment gives an event, observation, phenomenon for the student to explain or make a prediction about.

Criteria 2: Laboratory experiment gives a representation/model or asks student to construct a representation/model

Criteria 2a: Laboratory experiment gives a representation/model

Criteria 2b: Laboratory experiment asks student to construct a representation/model

Criteria 3: Laboratory experiment asks student to explain or make a prediction using the representation about the event, observation, or phenomenon.

Criteria 4: Laboratory experiment asks student to provide the reasoning that links the representation to their explanation or prediction.

## **SP3: Planning and Carrying Out Investigations**

Criteria 1: Laboratory experiment poses a scientific question, claim, or hypothesis to be investigated.

Criteria 2: Laboratory experiment asks student to describe or design an investigation, or identify the observations required to answer the question or test the claim or hypothesis.

Criteria 3: Laboratory experiment asks student to justify how their description, design or observations can be used to answer the question or test the claim or hypothesis.

## **SP4: Analyzing and Interpreting Data**

Criteria 1: Laboratory experiment gives a scientific question, claim or hypothesis to be investigated.

Criteria 2: Laboratory experiment gives a representation of the data (e.g., table or graph, or lists of observations) provided to answer the question or test the claim or hypothesis.

Criteria 2a: Laboratory experiment gives a representation of the data (e.g., table or graph, or lists of observations) provided to answer the question or test the claim or hypothesis.

Criteria 2b: Laboratory experiment has students generate a representation of the data (e.g., table or graph, or lists of observations) provided to answer the question or test the claim or hypothesis.

Criteria 3: Laboratory experiment gives an analysis of the data or asks student to analyze the data moving towards the goal of answering the research question/goal.

Criteria 3a: Laboratory experiment gives an analysis of the data

Criteria 3b: Laboratory experiment asks student to analyze the data

Criteria 4: Laboratory experiment asks student to interpret the results or assess the validity of the conclusions in the context of the scientific question, claim, or hypothesis.

## **SP5: Using Mathematical and Computational Thinking**

Criteria 1: Laboratory experiment gives an event, observation, or phenomenon.

Criteria 2: Laboratory experiment asks student to perform a calculation or statistical test, generate a mathematical representation, or demonstrate a relationship between parameters in order to obtain the correct answer.

Criteria 3: Laboratory experiment asks student to give a consequence or an interpretation (not a restatement) in words, diagrams, symbols, or graphs of their results in the context of the given event, observation or phenomenon.

## **SP6: Constructing Explanations and Engaging in Arguments from Evidence**

Criteria 1: Laboratory experiment gives an event, observation, or phenomenon.

Criteria 2: Laboratory experiment gives or asks student to make a claim based on the given event, observation, or phenomenon.

Criteria 2a: Laboratory experiment gives a claim based on the given event, observation, or phenomenon.

Criteria 2b: Laboratory experiment asks students to make a claim based on the given event, observation, or phenomenon.

Criteria 3: Laboratory experiment asks students to provide scientific principles or evidence in the form of data or observations to support the claim.

Criteria 4: Laboratory experiment asks student to provide reasoning about why the scientific principles or evidence support the claim.

## **SP7: Evaluate Information**

Criteria 1: Laboratory experiment gives, or student obtains, an excerpt from a conversation, article, student solution, or video that makes one or more assertions.

Criteria 2: Laboratory experiment gives a conclusion about the validity of the assertion(s) or asks student to make a conclusion about the validity of the assertion(s) or reconciliation of multiple assertions.

Criteria 2a: Laboratory experiment gives a conclusion about the validity of the assertion(s) or reconciliation of multiple assertions

Criteria 2b: Laboratory experiment asks student to make a conclusion about the validity of the assertion(s) or reconciliation of multiple assertions

Criteria 3: Laboratory experiment asks student to provide reasoning to support their conclusion(s) about the validity of the assertion(s) or reconciliation with data, observations, or scientific principles.

## **SP8: Defining Problems and Designing Solutions**

Students are asked to design/build something to serve as a function as a result of their investigation.

Criteria 1: Laboratory experiment gives an event, observation, or phenomenon, scenario, or model.

Criteria 2: Laboratory experiment asks students to identify the findings from their investigation that will be used in designing the solution and how they will be used.

Criteria 3: Laboratory experiment asks students to design or build a tangible product from the results of their investigation.

Criteria 4: Laboratory experiment asks students to discuss how they weighed or prioritized competing criteria, such as function, feasibility in production of design, and/or, in designing a solution.

## **SP9: Communicate Information**

Students are asked to present their findings of their experiments to an audience.

Criteria 1: Laboratory experiment gives an event, observation, phenomenon, scenario, or model.

Criteria 2: Laboratory experiment asks students to communicate their findings to an audience beyond the instructor, either another expert in chemistry or a general public audience.*

Criteria 2a: Laboratory experiment asks students to communicate their findings to another scientist.*

Criteria 2b: Laboratory experiment asks students to communicate their findings to a general public audience.

*This is a deviation from Carmel et. al. who previously just specified a general audience.
