# Supplementary material for: Bridging the Science Practices Gap: Analyzing Laboratory Materials for Their Opportunities for Engagement in Science Practices
Source: J Chem Educ. 2025 Feb 11;102(3):970–83. doi: 10.1021/acs.jchemed.4c00744 (PMC11905284; doi:10.1021/acs.jchemed.4c00744)
Supplement: Supplementary file 3 — ed4c00744_si_003.pdf [file ed4c00744_si_003.pdf]

Supporting Information

Bridging the science practices gap: Analyzing laboratory materials for their opportunities for engagement in science practices

Andrea L. Van Wyk[1^], Ardith Bhinu[1], Kimberley A. Frederick[2], Marya Lieberman[3], Renée S. Cole[1]

[1]Department of Chemistry, University of Iowa, Iowa City, IA 52242, USA
[2]Department of Chemistry, Skidmore College, Saratoga Springs, NY 12866, USA
[3]Department of Chemistry and Biochemistry, University of Notre Dame, Notre Dame, IN 46556, USA
^Present Address: Department of Chemistry and Physics, Drake University, Des Moines, IA 50311, USA

Changes in prompted opportunities to engage in science practices from pre-implementation and post-implementation laboratory materials.

Pre-implementation Opportunities for Engagement of Science Practices

To determine a baseline of the prompted opportunities present in faculty's laboratory materials prior to their participation in the MICRO project, the laboratory materials faculty initially submitted to represent their current instructional practices were analyzed for their opportunities to engage students in science practices. After applying the modified 3D-LAP to the 106 laboratory activities, we saw the following distribution presented in Figure S1.

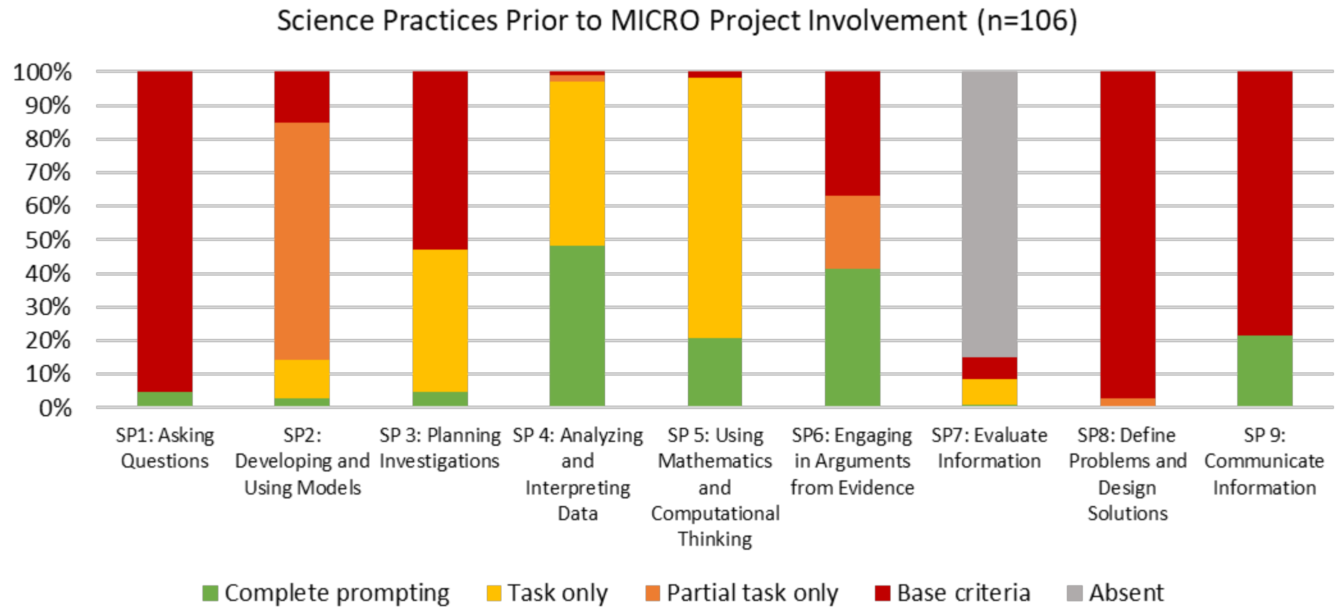

Figure S1.Results of 3D-LAP for participants' laboratory materials used prior to their involvement in the project.

We can see that the majority of laboratory experiments did not provide full opportunities to engage students in most of the science practices. We see higher percentages of laboratory experiments with fully met or almost met opportunities for *analyzing and interpreting data* (SP4)*, using mathematics and computational Thinking* (SP5)*,* and *engaging in arguments from evidence* (SP6) than for the other science practices. The higher emphasis on *analyzing and interpreting Data* (SP4) and *using mathematics and computational thinking* (SP5) was unsurprising as a lot of the content in analytical chemistry courses is focused on analyzing data and using various mathematical and computational approaches. *Engaging in arguments from evidence* (SP6) was often identified in post-laboratory questions of a laboratory activity. However, not all faculty had pre-laboratory and post-laboratory questions as elements of their laboratory activities. Overall, the materials that faculty were using prior to their involvement in the MICRO project had limited opportunities for students to engage in science practices.

MICRO labs vs. pre-implementation

During the semester of their involvement in the MICRO project, participants implemented 2-4 of the MICRO laboratory experiments in their course. While not the main goal of the MIRCO project, the MICRO laboratory experiments were designed with many of the science practices in mind. To determine how the MICRO laboratory experiments compared to faculty's current instructional practices we compared the opportunities to engage in science practices of the MICRO laboratory experiments (n=8) to the laboratory materials used by participants prior to their involvement in the MICRO project (n=106). The 3D-LAP results are provided in Figure

S2.

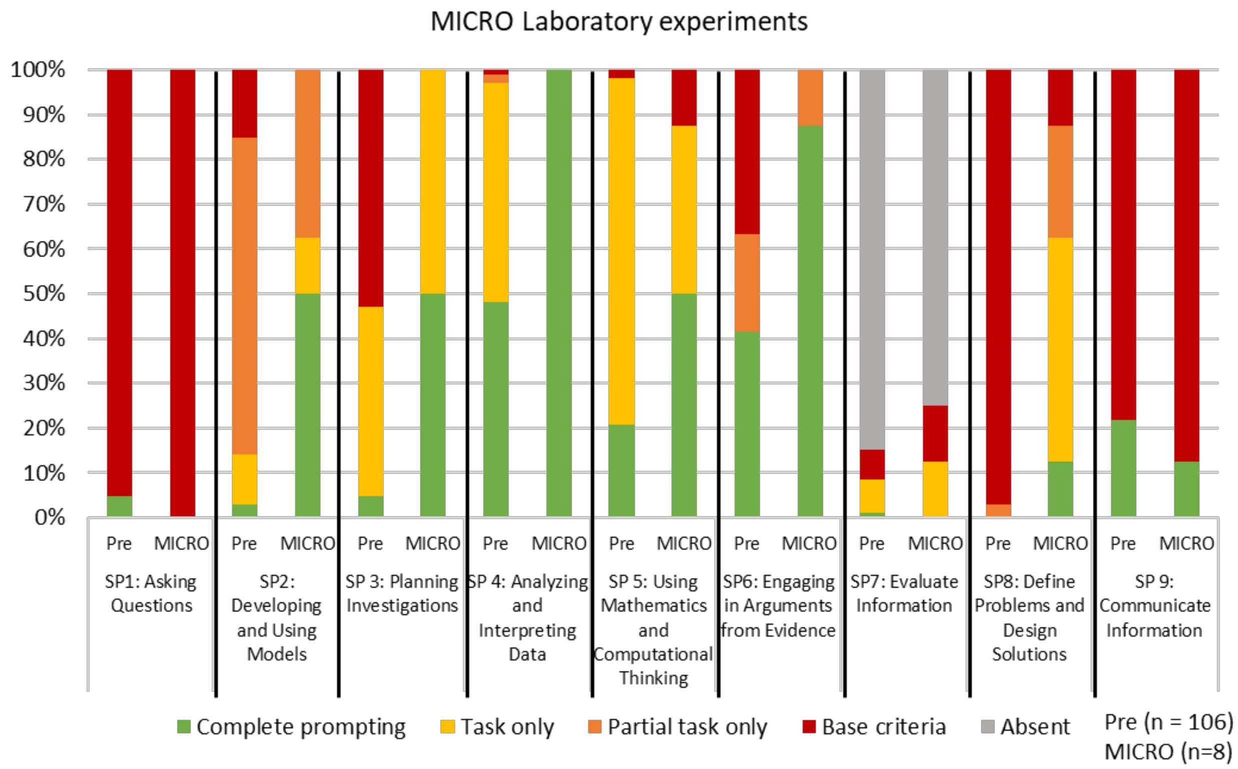

Figure S2.Comparison of the laboratory materials faculty used prior to their involvement in the project (n = 106 labs) and the MICRO laboratory materials (n = 8).

From these results we can see that for science practices 2-6 and 8 there are more complete opportunities to engage in these science practices in the MICRO laboratory experiments than faculty's current laboratory materials. Science practices 1, 7, and 9 didn't have much different between the MICRO laboratory experiments and faculty's current laboratory materials as they were practices that were not emphasized by the MICRO laboratory experiments or faculty's current laboratory materials. While not always providing full opportunities to engage in the targeted science practices, overall, the MICRO laboratory experiments have more of an emphasis on science practices 2-6 and 8 than the materials used by faculty prior to their involvement in the MICRO project. Since the MICRO project was structured to have faculty implement some of the MICRO laboratory experiments, they would be facilitating laboratory experiments where students have more opportunities to engage in science practices than their existing laboratory materials provided.

Post-implementation Opportunities for Engagement of Science Practices

To get a sense of how different faculty's instructional practices were the semester they were involved in the MICRO project we compared the opportunities to engage in science practices between the laboratory materials initially used by faculty and the laboratory materials faculty used the semester they were involved in the MICRO project. The results of applying 3D-LAP to each of these data sets are presented in Figure S3.

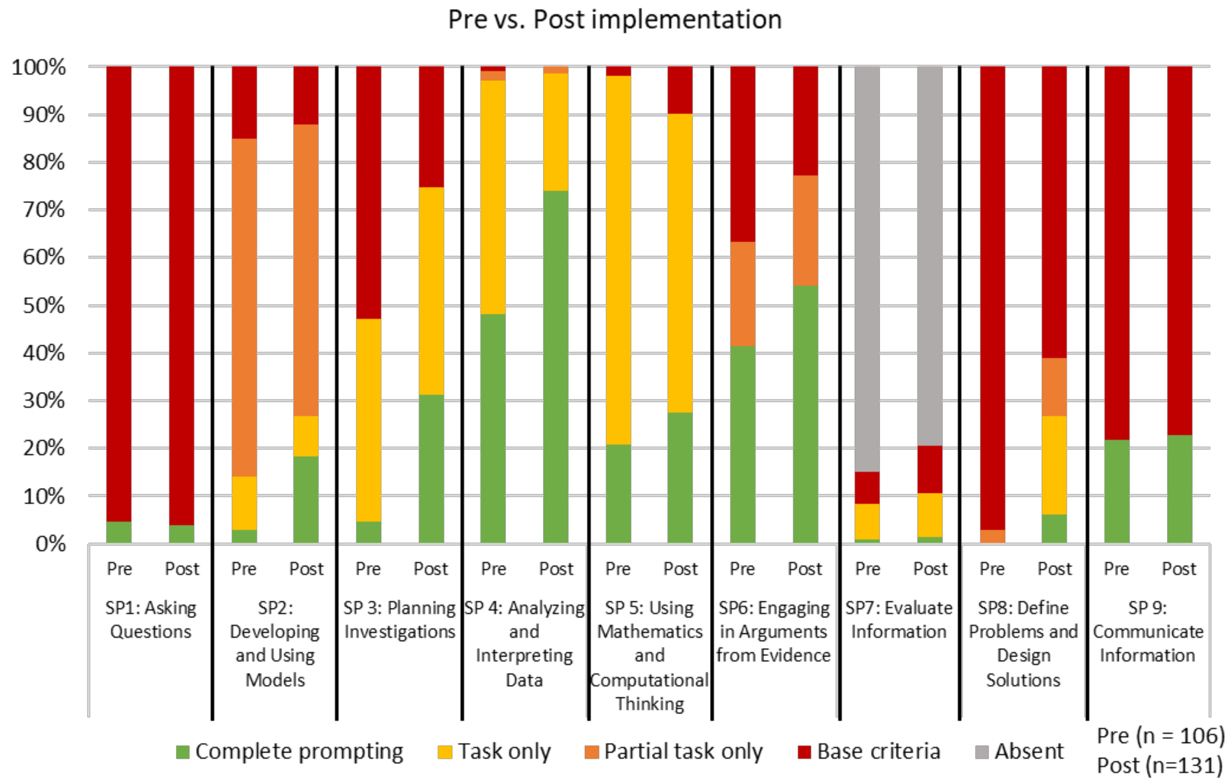

Figure S3. Comparison of laboratory materials representing faculty practices prior to involvement in the project (n = 106 labs) and then during their semester of involvement in the MICRO project (n= 131) for their opportunities to engage students in science practices.

From this data we see similar differences as were seen in the comparison of laboratory materials used by faculty initially and the MICRO laboratory experiments presented above in Figure S2. We see more complete opportunities to engage in science practices 2-6, and 8 for the laboratory materials faculty used during the semester they were involved in the MICRO project than they previously used. Additionally, we see minimal differences in science practices 1, 7, and 9. Since the MICRO laboratory materials emphasized more completed opportunities to engage in science practices 2-6 and 8 and had minimal opportunities to engage in science

practices 1, 7, and 9, we expected to see these differences as faculty implemented 2-4 MICRO laboratory experiments into their courses.

With the incorporation of 2-4 of the MICRO experiments into their curriculum faculty got some exposure to facilitating laboratory experiments with more opportunities to engage students in science practices. In the MICRO workshop we also spent some time talking about science practices, designing learning objectives to emphasize science practices, and ways to embed opportunities to engage students in science practices into laboratory experiments. Because of this we sought to determine if faculty made any changes to the other laboratory experiments that they implemented other than the 2-4 MICRO laboratory experiments to increase their opportunities to engage students in some of the science practices. To do this we compared the laboratory materials faculty used prior to involvement in the project (n = 106 labs) to the materials they used during their involvement in the project except we removed the MICRO laboratory experiments they implemented (resulting in n = 83 labs). The results of this

comparison are presented in Figure S4.

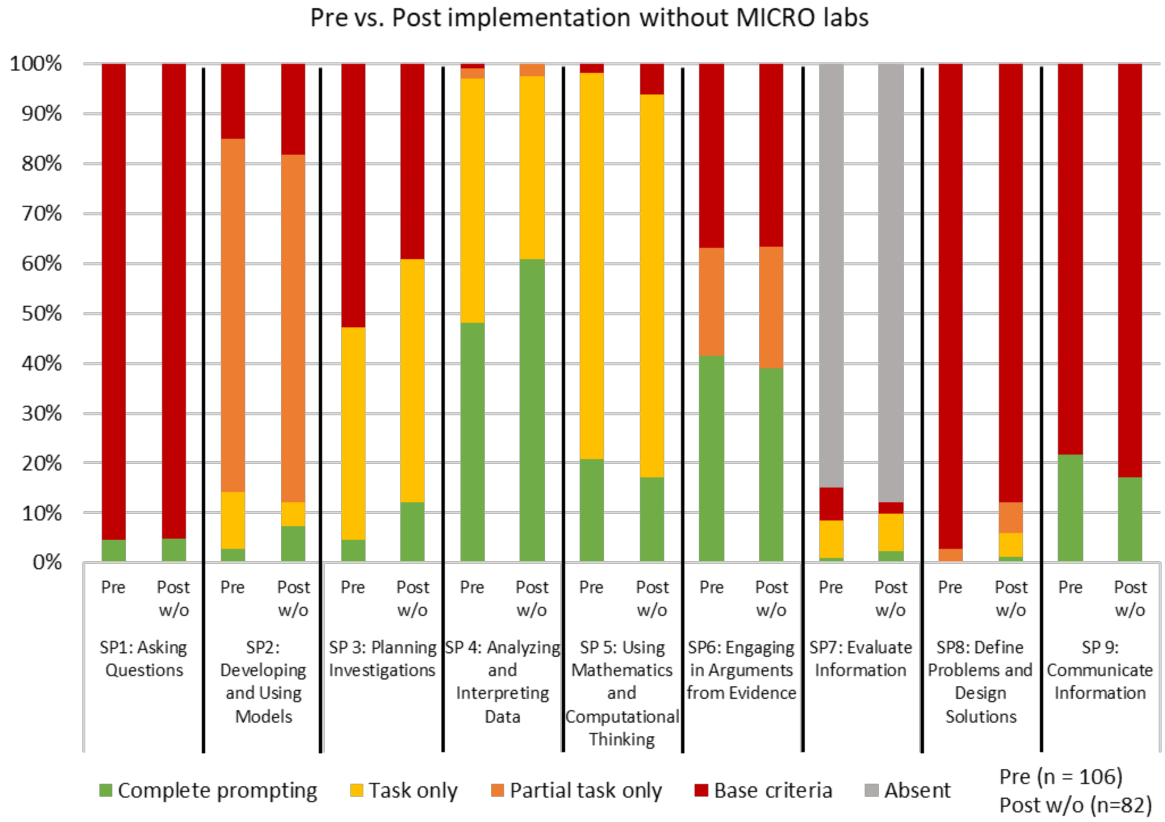

Figure S4. Comparison of laboratory materials representing faculty practices prior to involvement in the project (n = 106 labs) and then during their semester of involvement in the MICRO project with the MICRO laboratory experiments they implemented excluded (n= 83) for their opportunities to engage students in science practices.

In comparing these two data sets, we can see that there are small differences for the science practices *planning and carrying out investigations* (SP3) and *analyzing and interpreting data* (SP4). For the rest of the science practices there is little difference between the two data sets. This trend held true when comparing materials for each instructor individually. This indicates that there were no significant changes between faculty's pre-implementation laboratory materials and their post-implementation laboratory materials in terms of prompted opportunities to engage in many of the science practices.
